# Prevention of Birch Pollen-Related Food Allergy by Mucosal Treatment with Multi-Allergen-Chimers in Mice

**DOI:** 10.1371/journal.pone.0039409

**Published:** 2012-06-29

**Authors:** Elisabeth Hoflehner, Karin Hufnagl, Irma Schabussova, Joanna Jasinska, Karin Hoffmann-Sommergruber, Barbara Bohle, Rick M. Maizels, Ursula Wiedermann

**Affiliations:** 1 Institute of Specific Prophylaxis and Tropical Medicine, Center for Pathophysiology, Infectiology and Immunology, Medical University of Vienna, Vienna, Austria; 2 Department of Pathophysiology and Allergy Research, Center for Pathophysiology, Infectiology and Immunology, Medical University of Vienna, Vienna, Austria; 3 Institute of Immunology and Infection Research, University of Edinburgh, Edinburgh, United Kingdom; Centre de Recherche Public de la Santé (CRP-Santé), Luxembourg

## Abstract

**Background:**

Among birch pollen allergic patients up to 70% develop allergic reactions to Bet v 1-homologue food allergens such as Api g 1 (celery) or Dau c 1 (carrot), termed as birch pollen-related food allergy. In most cases, specific immunotherapy with birch pollen extracts does not reduce allergic symptoms to the homologue food allergens. We therefore genetically engineered a multi-allergen chimer and tested if mucosal treatment with this construct could represent a novel approach for prevention of birch pollen-related food allergy.

**Methodology:**

BALB/c mice were poly-sensitized with a mixture of Bet v 1, Api g 1 and Dau c 1 followed by a sublingual challenge with carrot, celery and birch pollen extracts. For prevention of allergy sensitization an allergen chimer composed of immunodominant T cell epitopes of Api g 1 and Dau c 1 linked to the whole Bet v 1 allergen, was intranasally applied prior to sensitization.

**Results:**

Intranasal pretreatment with the allergen chimer led to significantly decreased antigen-specific IgE-dependent β-hexosaminidase release, but enhanced allergen-specific IgG2a and IgA antibodies. Accordingly, IL-4 levels in spleen cell cultures and IL-5 levels in restimulated spleen and cervical lymph node cell cultures were markedly reduced, while IFN-γ levels were increased. Immunomodulation was associated with increased IL-10, TGF-β and Foxp3 mRNA levels in NALT and Foxp3 in oral mucosal tissues. Treatment with anti-TGF-β, anti-IL10R or anti-CD25 antibodies abrogated the suppression of allergic responses induced by the chimer.

**Conclusion:**

Our results indicate that mucosal application of the allergen chimer led to decreased Th2 immune responses against Bet v 1 and its homologue food allergens Api g 1 and Dau c 1 by regulatory and Th1-biased immune responses. These data suggest that mucosal treatment with a multi-allergen vaccine could be a promising treatment strategy to prevent birch pollen-related food allergy.

## Introduction

One of the most common type I pollionosis is caused by the airborne allergens of birch pollen (BP). In Europe, more than 70% of BP-allergic patients develop an immediate hypersensitivity reaction against pollen-related food allergens, termed as birch pollen-related food allergy (BPRFA) and clinically manifested as oral allergy syndrome (OAS). IgE antibodies specific for Bet v 1, the major BP allergen, cross-react with epitopes of homologous food allergens such as Mald1 (apple), Cora1 (hazelnut), Api g 1 (celery), or Dau c 1 (carrot) [1], [2]. Due to this cross-reactivity, Bet v 1-specific IgE can induce hypersensitivity reactions towards these food allergens. The symptoms of the BPRFA are usually restricted to the oral cavity and can range from swelling and itching of lips, tongue, soft palate and pharynx to systemic reactions such as urticaria, asthma or even anaphylaxis [3], [4]. Most of these patients also display food induced symptoms outside the BP season, indicating that homologous food allergens provide a perennial boost of BP-specific immune responses [5].

For BP mono-sensitized individuals common specific immunotherapy (SIT) is well established and is regarded as a successful therapy. However, for treatment of patients with multiple sensitivities or BPRFA, SIT has low efficacy and is associated with an increased risk of anaphylactic side-effects [1], [6], [7]. Improving this treatment could either be achieved by the application of well defined recombinant single allergens or a mixture thereof, or allergen peptides according to the patient T cell recognition pattern. Additionally, exploiting different routes of vaccination, e.g. changing the subcutaneous to a less invasive administration via the mucosa (i.e. oral, nasal, sublingual) could improve the efficacy of this treatment [8].

We previously demonstrated that mucosal administration of recombinant allergens prevented allergic sensitization in mono-sensitized mice [9]. In poly-sensitized mice, however, application of a mixture of recombinant antigens did not efficiently elicit protective effects [8], [10]. More recently, we demonstrated that mucosal application of either a multi-peptide construct, covering the immunodominant T cell epitopes of the major birch and grass pollen allergens, or a multi-allergen chimer, consisting of the scaffold allergen Bet v 1 in its native conformation anchoring two or more immunodominant peptides from major grass pollen allergens, prevented multi-sensitization against these allergens [8], [10].

In the current study we established a model of BPRFA in poly-sensitized mice to validate the protective effects of mucosal treatment with a respective chimer. For this purpose we designed a pollen-food-allergen chimer consisting of Bet v 1, acting as a potent tolerogen, fused with additional immunodominant peptides of its homologous food allergens Api g 1 from celery and Dau c 1 from carrot. Our data provide evidence for the efficacy and underlying mechanisms of mucosal treatment with this chimer in preventing local and systemic Th2 immune responses in poly-sensitized mice.

## Methods

### Animals

Female 7-week-old BALB/c mice (n = 12 per group) were obtained from Charles River (Sulzfeld, Germany). All experiments were repeated 3 times.

### Ethics Statement

The animal studies were performed according to institutional guidelines for animal use and care. The study was approved by the Animal Experimentation Ethics Committee of the Medical University of Vienna and the Ministry of Science and Research (GZ 66.009/229-BrGT/2005; GZ 66.009/35-II/10b/2010).

### Antigens and Antibodies

Recombinant Bet v 1.010, Api g 1.0101 and Dau c 1.0103 were obtained from Biomay AG (Vienna, Austria). Birch pollen (BP) from *Betula verrucosa* was purchased from Allergon (Välinge, Sweden ), and protein extracts of BP, celery (*Apium graveolens) and* carrot (*Daucus carota*) were prepared as previously described [11], [12].

Anti-TGF-β, anti-IL-10R and anti-CD25 blocking antibodies were produced in house at the University of Edinburgh, UK (and provided by R. Maizels). Rat IgG isotype control antibody was used (Sigma-Aldrich).

### Epitope Mapping Studies

For T cell epitope mapping a panel of 48 peptides of Api g 1 and 48 peptides of Dau c 1 were used (provided by B. Bohle). Spleen cell suspensions from Api g 1 or Dau c 1 immunized mice were incubated with 5 µg/well of each of the peptides, which overlapped for three amino acids (neighbours sharing nine residues) spanning the whole amino acid sequence of the respective antigens. Proliferative responses were measured according to previous description [12].

### Construction of the Birch Pollen–food–chimer Expression Plasmid

Complementary templates from Api g 1 (Genbank Access number: Z48967) and Dau c 1 (Genbank Access number: Z84376) respectively, were used to amplify the identified immunodominant encoding regions by PCR (cDNA templates were provided by K. Hoffmann-Sommergruber). Api g 1-T cell epitope specific primers were designed including *Nco*I and *Eco*RI restriction sites (Api g 1 fwd 5′-CATGCCATGGATGGAGTTAACAAGGAG-3′, Api g 1 rev 5′-ATGAATTCAACATGGTTTTCAATGGA-3′; restriction sites underlined); Dau c 1-T cell epitope specific primers were designed including *Hind*III and *Xho*I restriction sites (Dau c 1 fwd 5′-ACCAAGCTTGCCGTGGTTCCTGAAGAG-3′, Dau c 1 rev 5′-CCGCTCGAGTTAATTAGCAATGAGGTAGGC-3′).

For construction of the Api g 1-Bet v 1-Dau c 1-chimer we ligated the PCR amplicons of Api g 1 and Dau c 1 into the respective restriction sites on the 5′- and 3′-end of a pHis Parallel 2 - Bet v 1 plasmid, equipped with a hexahistidyl (6×His) affinity tag as previously described [8]. Subsequent DNA sequence analysis (GATC Biotech, Konstanz, Germany) verified correct sequences and the integrity of open reading frame.

### Expression, Purification and Refolding of Recombinant Api g 1-Bet v 1-Dau c 1-chimer

The Api g 1-Bet v 1-Dau c 1-expression plasmid was transformed into electrocompetent BL 21(DE3)plysS *E. coli* cells (Invitrogen, NV Leek, Netherlands) and grown in LB/1 mM ampicillin medium at 37°C under vigorous shaking until an optical density (OD_600_) of 0.7. Expression was induced by adding 0.001 mol/L isopropyl β-D-thiogalactopyranoside (IPTG) and incubation continued for additional 3 hours before harvesting.

6×His-tagged r Api g 1-Bet v 1-Dau c 1-chimeric protein was produced in inclusion bodies and therefore purified from *E. coli* lysate under denaturing conditions. Cells were solubilized in denaturating lysis buffer (8 mol/L urea, 0.1 mol/L NaH_2_PO_4_, 0.01 mol/L tris, pH 8.0) and purified by nickel nitrilotriacetic acid affinity column (GE Healthcare, Uppsala, Sweden).

Purified protein fractions were monitored by sodium dodecyl sulphate-polyacrylamide gel electrophoresis (SDS-PAGE), pooled and refolded by stepwise dialysis against 0.02 mol/L NaPO_4_, pH 8.0 while gradually reducing urea concentration from 6 mol/L to 0 mol/L. After a final dialysis against 0.01 mol/L NaPO_4_, pH 7.2, Api g 1-Bet v 1-Dau c 1-chimer was lyophilized and stored at −20°C.

Concentration of bacterial endotoxins was determined using the *Limulus amebocyte lysate* assay (QCL-1000, Cambrex, Walkersville, MD, USA) according to the useŕs manual. Endotoxins were removed using the EndoTrap Blue affinity column (Profos, Regensburg, Germany) according to the manufacture’s instructions. Protein concentration was determined using the bicinchronic acid protein assay (Pierce, Rockford, IL, USA).

### Protein Analysis

Physiochemical identification and characterization of the recombinant construct was done by preparative HPLC and mass spectrometry (piCHEM, Graz, Austria), and secondary structure by far UV light circular dichroism spectroscopy (CD), as previously described [8].

Immunological characterization was done by immunoblot analysis using a monoclonal mouse anti-Bet v 1 IgG antibody (BIP1, 1/10), sera from 3 BP allergic patients with concomitant BPRFA containing Api g 1-, Dau c 1-, and Bet v 1-specific IgE (1/5) or mouse sera from a chimer-tolerized, poly-sensitized mouse (1/5). For detection secondary rat anti-mouse IgG1 antibody (1/500; BD Pharmingen, San Diego, CA, USA) followed by an alkaline phosphatise-conjugated goat anti-rat IgG (1/2000; Santa Cruz Biotechnology, Santa Cruz, CA, USA), or alkaline phosphatase-conjugated mouse anti-human IgE (1/1000; Pharmingen) antibodies and NBT/BCIP substrate mixture were used. Negative controls with sera from untreated mice and non-allergic patients, or buffer control were run in parallel.

### Animal Treatment

#### Allergy prevention in mono-sensitized mice with either rBet v 1, rApi g 1 or rDau c 1

Mono-sensitization was performed by 3 intraperitoneal (i.p.) injections (day 22, 36, 50) of 5 µg per Bet v 1, Api g 1 or Dau c 1 adsorbed to aluminium hydroxide (Al(OH)_3_; Serva, Heidelberg, Germany) in 14 day intervals, adapted from Hufnagl et al. [13]. For prevention of allergic sensitization 10 µg of either of the allergen Bet v 1, Api g 1 or Dau c 1 were intranasally (i.n.) applied in 30 µl of 0.9% NaCl 3 times in 7 day intervals (day 0, 7, 14), prior to mono-sensitization, adapted from Wiedermann et al. [12]. Samples were taken one week after the last treatment (day 57).

#### Allergy prevention in poly-sensitized mice with a mixture of all three allergens

Poly-sensitization was performed by applying a mixture of Bet v 1, Api g 1 and Dau c 1, 5 µg each adsorbed to Al(OH)_3_ as described above. For prevention of poly-sensitization mice were i.n. pretreated with a mixture of 10 µg each of Bet v 1, Api g 1 and Dau c 1, in 30 µl of 0.9% NaCl. Allergy prevention and poly-sensitization protocols were adapted from Hufnagl et al. [10].

#### Allergy prevention with the birch pollen-food chimer in poly-sensitized mice

**Figure 1:**
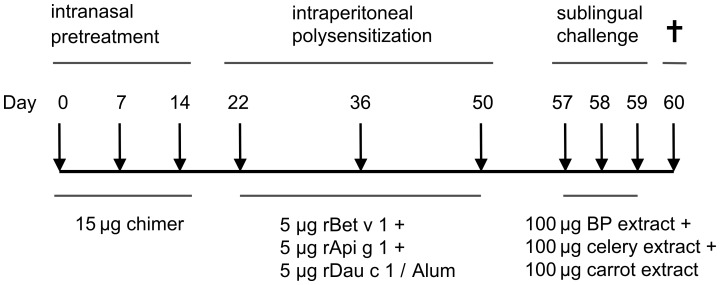
Experimental design. Mice were intranasally pretreated with either the chimer (chimer-treat) or sham-treated (poly-sens) 3 times in 7 days intervals followed by 3 intraperitoneal sensitizations with a mixture of rBet v 1, rApi g 1 and rDau c 1 in 2 weeks intervals. Thereafter, mice were sublingually challenged with a mixture of BP, celery and carrot extracts on 3 consecutive days.

Intranasal pretreatment with the BP-food chimer was performed by using 15 µg of the chimer in 30 µl of 0.9% NaCl per application (adapted from Wild et al. [8] ) as described above, prior to poly-sensitization. Control mice were i.n. sham-treated with 30 µl of 0.9% NaCl prior to poly-sensitization. One week after the last i.p. immunization, mice were sublingually (s.l.) challenged with a mixture of BP extract, carrot extract and celery extract, by applying 100 µg each in 15 µl of 0.9% NaCl with a pipette under the tongue of the mice. To prevent swallowing of the extracts mice were fixed in the scruff during and until 20 seconds after treatment. Mice were challenged on 3 consecutive days in 24 hour intervals (day 57, 58, 59). 24 hours after the last treatment mice were sacrificed (day 60) (Fig. 1).

#### In vivo application of neutralizing antibodies

In some experiments, i.n. chimer-pretreated and poly-sensitized mice were i.p. injected with 0.5 mg of blocking antibodies. Anti-CD25 was applied prior poly-sensitization (day 15) (adapted from Leech et al. and Wilson et al.) [14], [15], anti-TGF-β before s.l. challenge (day 56) (adapted from Taher et al. [16]), and anti-IL-10R was injected before poly-sensitization (day 19) and before s.l. challenge (day 56) (adapted from Taher et al., Leech et al. and Wilson et al. [14], [15], [16]). Pretreated and poly-sensitized control mice were sham-treated with 0.5 mg isotype control antibodies.

### Sampling

Blood samples were taken before treatment and on the day of sacrifice by tail bleeding. Sera were collected and stored at −20°C until analysis. On the day of sacrifice cell suspensions from spleen, cervical lymph nodes (CLN) and NALT were prepared as described [12], [17], [18]. Additionally, sublingual tissues (SLT) and buccal mucosa (BM) were prepared by excising the whole mandible from the head and dissecting the cheek skin and the tongue with the floor of the mouth (SLT). The cheek skin was stretched and mucosal tissue was scraped off with a scalpel and collected in RNA*later* buffer (Qiagen, Valencia, CA) for RNA isolation. SLT was separated from the tongue and stored in RNA*later* buffer as well [19].

### Allergen-specific Antibody Levels and Total IgA in Serum

Microtiter plates (Nunc, Roskilde, Denmark) were coated with each of the recombinant allergens Bet v 1, Api g 1 or Dau c 1 (5 µg/mL) prior to incubation with sera in dilutions of 1/500 for antigen-specific IgG2a and 1/10 for antigen-specific IgA detection. Rat anti-mouse IgG2a or IgA antibodies (1/500, Pharmingen) were used, followed by peroxidase-conjugated mouse anti-rat IgG antibody (1/2000, Jackson Immuno Lab, West Grove, PA) [12]. Results show the OD values after subtraction of baseline levels from pre-immune sera.

For determination of total IgA levels in sera, microtiter plates (Nunc) were coated with rat anti-mouse IgA (1/250, clone C10-3, Pharmingen) and incubated with 1/100 diluted sera. For detection biotinylated anti-mouse IgA (1/1000, clone C 10-1, Pharmingen) antibody, streptavidin (1/10000) and ABTS substrate were used.

Results are shown in ng/mL after subtraction of baseline levels of pre-immune sera.

### Rat Basophil Leukemia Cell Mediator Release Assay (RBL Assay)

For measurement of functional allergen-specific IgE, rat basophil leukemia (RBL) cells (RBL-2H3 cell line, ATCC, No. CRL-2256) were incubated with sera obtained from pretreated and poly-sensitized mice at dilutions of 1/10, 1/100 and 1/300. Degranulation of RBL cells was induced by adding 0.03 µg of Bet v 1, Api g 1 or Dau c 1 diluted in 100 µl Tyrodés buffer. Supernatants were analyzed for antigen-specific IgE-dependent β-hexosaminidase activity as previously described [13].

### Cytokine Production

IL-4, IL-5, IFN-γ and TGF-β production was measured in spleen (5×10^6^ cells/well), CLN (5×10^5^ cells/well) and NALT suspensions (5×10^5^ cells/well) incubated for 48 hours with each allergen (15 µg/well) as described [17]. Levels of IL-4, IL-5 and TGF-β were measured with ELISA kits (eBioscience, San Diego, CA, USA), IFN-γ levels were measured as previously described [20]. All cytokine levels are shown in pg/mL after subtraction of baseline levels of unstimulated cultures.

### Magnetic Sorting of CD4^+^CD25^+^ Tregs and CD4^+^CD25^−^ T Effector Cells

CD4^+^CD25^−^ T effector cells (Teff) and CD4^+^CD25^+^ Treg cells were isolated from pooled spleen cells of chimer-pretreated or control poly-sensitized mice, with the MACS CD4^+^CD25^+^ Treg isolation kit (Miltenyi Biotec, Bergisch Gladbach, Germany), according to the manufacturer’s protocol. The purity of sorted cells was acquired by flow cytometry using a BD FACSCalibur (BD Biosciences Pharmingen) and analysed by FlowJo software.

### Treg Suppression Assay

CD4^+^CD25^−^ Teff cells (5×10^4^) were cocultured in U-bottom 96-well plates with CD4^+^CD25^+^ Treg cells in various ratios (1/2, 1/8, 1/16). The cells were stimulated with 1 µg/mL anti-mouse CD3 (eBioscience) and 5×10^4^ irradiated (3000 rad) splenocytes at 37°C for 3 days. For the last 16 hours of culture, cells were pulsed with 0.5 µCi ^3^H-thymidine/well (Perkin-Elmer, Wellesley, MA, USA), harvested and proliferative responses were measured by scintillation counting (1450 Microbeta Liquid Scintillation and Luminescence counter, Perkin-Elmer). Results are expressed as absolute counts per minute (cpm). The percent suppression mediated by Treg cells was calculated by the following formula: [(cpm of Teff alone – cpm of Teff treated with Treg)/cpm of Teff cells alone]*100.

### Quantification of mRNA Expression by Real-time RT-PCR

Total RNA from NALT was isolated from equal pooled cell suspensions. Total RNA extracted from RNA*later*-stabilized, pooled SLT and BM was homogenized in liquid nitrogen prior to purification by using RNeasy Minikit combined with DNase digestion (RNase-free DNase Set, Qiagen). The concentration of extracted RNA was measured by NanoDrop ND-1000 spectrophotometer (PeqLab, Erlangen, Germany), RNA probes were standardized and then reverse-transcribed into cDNA using iScript cDNA Synthesis Kit (Bio-Rad Laboratories, Hercules, CA, USA).

Gene expression was determined by quantitative real-time RT-PCR using LightCycler® FastStart kit with TaqMan® or CYBRGreen according to the manufacturer’s instructions (Roche, Mannheim, Germany) on a LightCycler® instrument 1.2 (Roche). For quantification of TGF-β, IL-10 and Foxp3 mRNA, pre-designed TaqMan® assays were used. Data are presented as the relative ratio of the target genes to the housekeeping gene *5-aminolevulinic acid synthase 1* (*Alas1*) (Universal ProbeLibrary (UPL) probe #64, Roche) [21].

### Statistics

Data are expressed as means ± SEMs from 3 independent experiments. For statistical analysis *p* values <0.05 were defined significant. Pair-wise comparison of sham-treated sensitized versus pretreated groups was performed by using the Mann-Whitney *U-*test and one-way ANOVA-test.

## Results

### Intranasal Pretreatment with Bet v 1, Api g 1 and Dau c 1 Alone or as a Mixture does not Suppress Immune Responses Against All 3 Allergens

**Figure 2:**
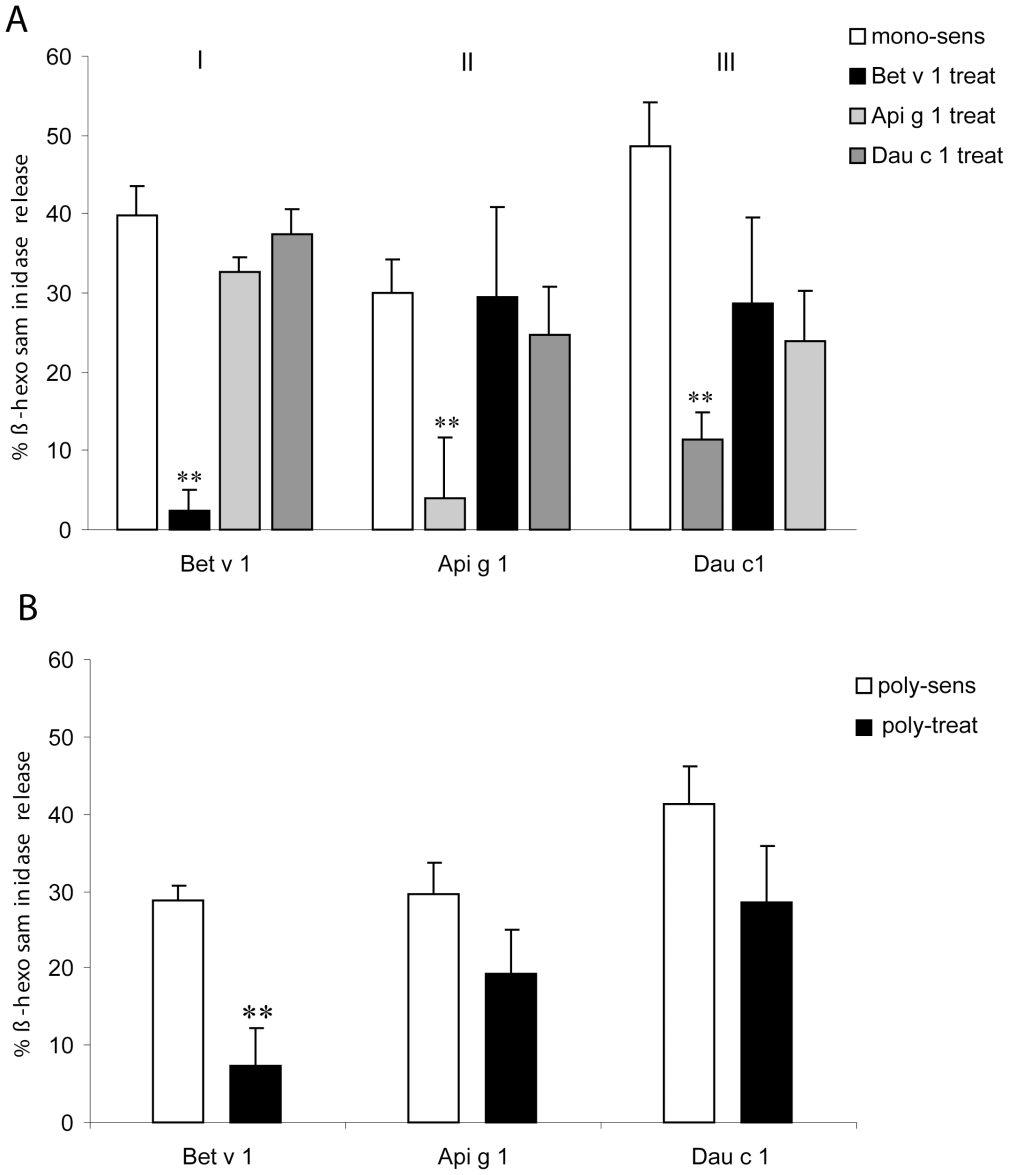
IgE-dependent allergen-specific basophil degranulation by sera. (**A**) ß-hexosaminidase release from (**I.**) rBet v 1-, (**II.**) Api g 1- or (**III.**) Dau c 1-sensitized mice; each group was i.n. pretreated with rBet v 1 (black bars), rApi g 1 (light-grey bars), rDau c 1 (dark-grey bars) or sham-treated (white bars). (**B**) ß-hexosaminidase release from mice i.n. pretreated with a mixture of rBet v 1/rApi g 1/rDau c 1 (black bars) compared with poly-sensitized controls (white bars). **p<0.01.

Mucosal application of Bet v 1 prior to intraperitoneal polysensitization significantly reduced serum IgE responses as measured by IgE-induced basophil degranulation to Bet v 1, but not to Api g 1 or Dau c 1 (Fig. 2A). Similarly, mucosal application of Api g 1 or Dau c 1 prior to poly-sensitization showed significantly decreased IgE-induced β-hexosaminidase release only for the respective allergen (Fig. 2A).

In poly-sensitized mice, mucosal pretreatment with a mixture of all 3 allergens significantly reduced basophil degranulation to Bet v 1, but not to the homologous food allergens (Fig. 2B).

### Construction and Characterization of the Birch Pollen-food Allergen Chimer

**Figure 3:**
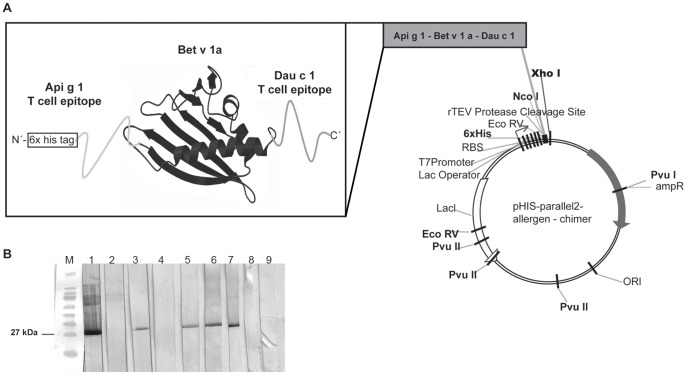
Construction and characterization of the pollen-food-chimer. (**A**) Design of the *pHis-parallel 2*-chimer composed of Bet v 1 protein, flanked by peptides from Api g 1 and Dau c 1. (**B**) Immunoblot: IgE binding to the chimer of sera from chimer-pretreated/poly-sensitized mice (lane 1) and of BP allergic patients with BPRFA (lane 5, 6, 7), and of Bet v 1 monoclonal antibody (lane 3). Negative controls sera from untreated mouse and non-allergic patient, or buffer control were run in parallel (lane 2, 4, 8. 9).

Based on previous T cell epitope mapping experiments with Api g 1 and Dau c 1 the immunodominant regions of these allergens were selected for designing the Bet v 1-food allergen chimer. In the case of Dau c 1 the immunodominant peptide detected in mice is also a major T cell epitope in allergic patients [22]. The immunodominant T cell epitope from Api g 1 (DGVNKEALTFDYSVIDGDILLGFIESIENHV, peptide 27) including a 6×His-tag was inserted at the N-terminus of the Bet v 1.0101 encoding sequence. At the Ć-terminus the immunodominant T cell epitope of Dau c 1 (AVVPEENIKFADAQNTALFKAIEAYLIAN, peptide 47) was added (Fig.3A). Correct insertion of the templates was checked by sequence analysis.

After purification and refolding of the chimer with a theoretical mass of 27,6 kD, endotoxin levels of <0,05 EU/µg of purified protein were measured, which corresponds to baseline levels of commercially available proteins [9].

Secondary structure elements of the chimer, analyzed by CD spectra, were in good agreement with the spectra obtained from rBet v 1 [8]. Only minimal variations due to the added peptides (data not shown) were observed. Immunoblot analysis confirmed that the conformation of Bet v 1 remained unchanged after linkage of the peptides. The chimer was recognized by Bet v 1-specific monoclonal antibody (BIP1, lane 3) and by IgE from sera of 3 BP allergic patients with a BPRFA (lane 5, 6, 7) as well as IgE from sera of a chimer-treated poly-sensitized mouse (lane 1). Negative controls did not elicit any IgE binding to the chimer (lane 2, 8, 4, 9) (Fig. 3B).

### Intranasal Pretreatment with the BP-food-chimer Suppressed Humoral and Cellular Immune Responses in Poly-sensitized Mice

#### Antibody responses

**Figure 4:**
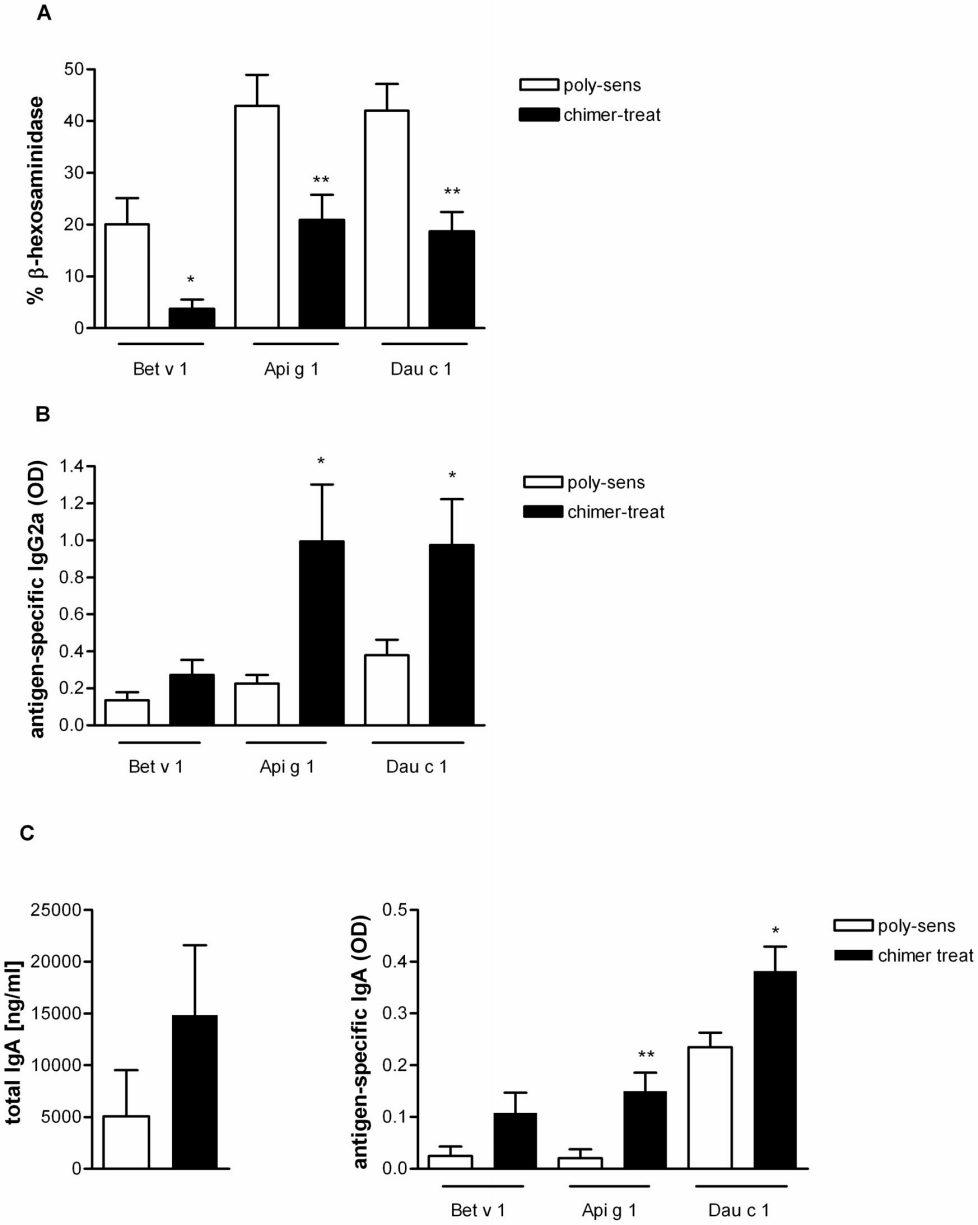
Antigen-specific cellular and humoral responses in mice pretreated with the chimer and poly-sensitized control mice. (**A**) IgE-mediated basophil degranulation. (**B**) Allergen-specific IgG2a antibodies in sera. (**C**) Total and antigen-specific IgA antibodies in sera. Chimer-pretreated mice (black bars); poly-sensitized control mice (white bars). *p<0.05, **p<0.01.

Intranasal pretreatment with the chimer significantly reduced IgE-mediated basophil degranulation to all three allergens in comparison to the untreated poly-sensitized group (Fig. 4A). Moreover, Api g 1-, Dau c 1- and Bet v 1-specific IgG2a antibody production was enhanced, indicating a shift towards Th1 responses (Fig. 4B). Additionally, pretreatment with the chimer increased serum levels of total and allergen-specific IgA antibodies in comparison to poly-sensitized controls (Fig. 4C).

#### Cytokine production

**Figure 5:**
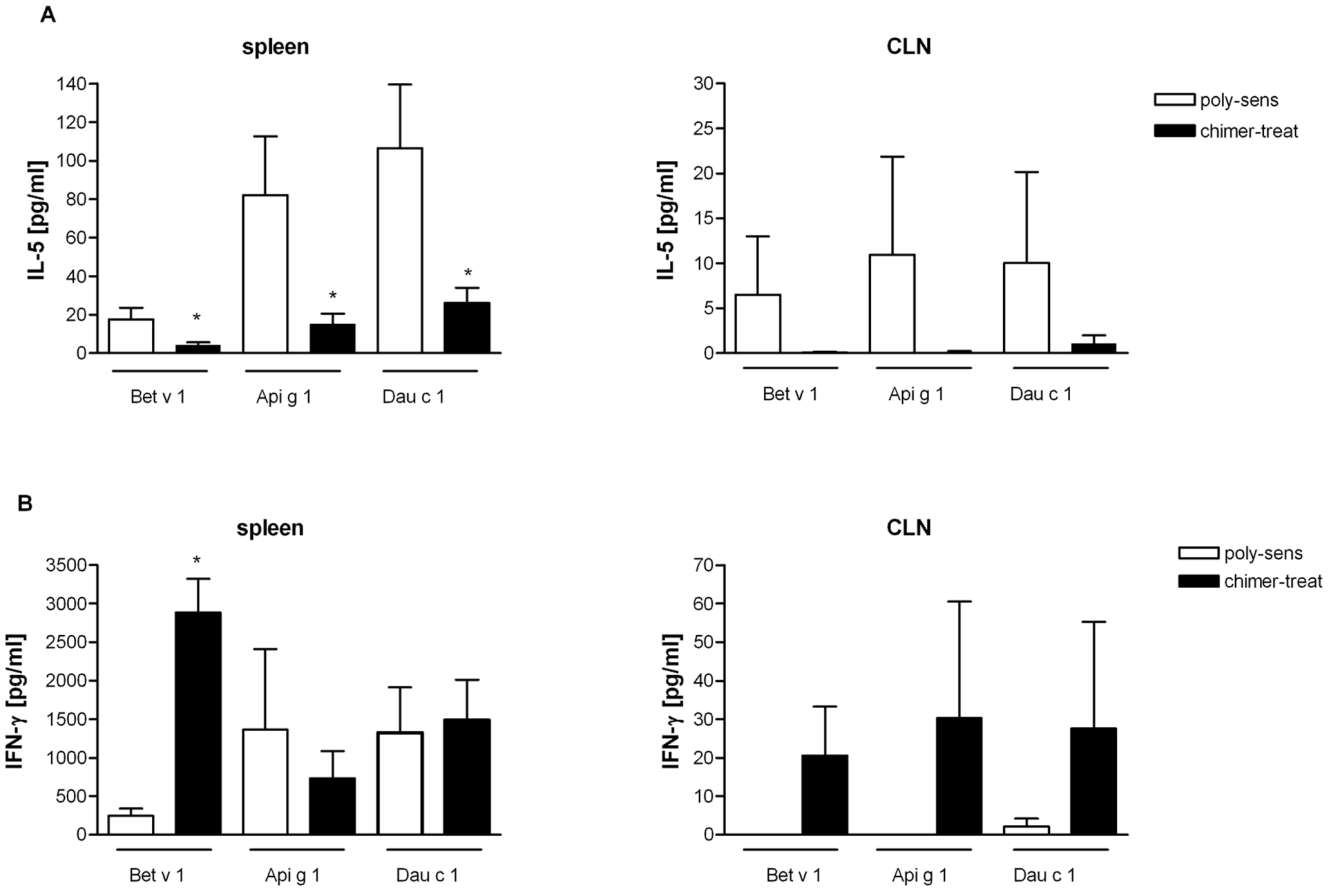
Antigen-specific cytokine production in mice pretreated with the chimer and poly-sensitized mice. (**A**) IL-5 levels and (**B**) IFN-γ levels in supernatants of spleen and cervical lymph nodes (CLN) cell cultures after antigen stimulation. Chimer-pretreated mice (black bars); poly-sensitized control mice (white bars). *p<0.05, **p<0.01.

Pretreatment with the chimer significantly decreased IL-5 levels in supernatants of allergen restimulated spleen and CLN cell cultures (Fig. 5A). Additionally, the IgE switching factor IL-4 was markedly reduced for all 3 allergens in spleen cell cultures of chimer treated mice compared to poly-sensitized controls: IL-4 (pg/mL): polysens: Bet v 1-restimulated: 14,35±22,60; Api g 1-restimulated: 43,92±53,84; Dau c 1-restimulated: 58,87±64,83; chimer-treat: Bet v 1-restimulated: 5,06±7,45; Api g 1-restimulated: 7,61±8,14**; Dau c 1-restimulated: 11,89±13,70**; **p<0,01.

IFN-γ production in spleen cell cultures was significantly enhanced after Bet v 1 - but not after Api g 1 or Dau c 1 - stimulations compared to poly-sensitized controls (Fig. 5B). However, in the CLN (Fig. 5B), and also in the NALT (data not shown) cultures, IFN-γ production was significantly increased in the pretreated mice after re-stimulation with all three allergens.

### Prevention of Allergic Polysensitization with the Chimer is Associated with Regulatory Mechanisms

**Figure 6:**
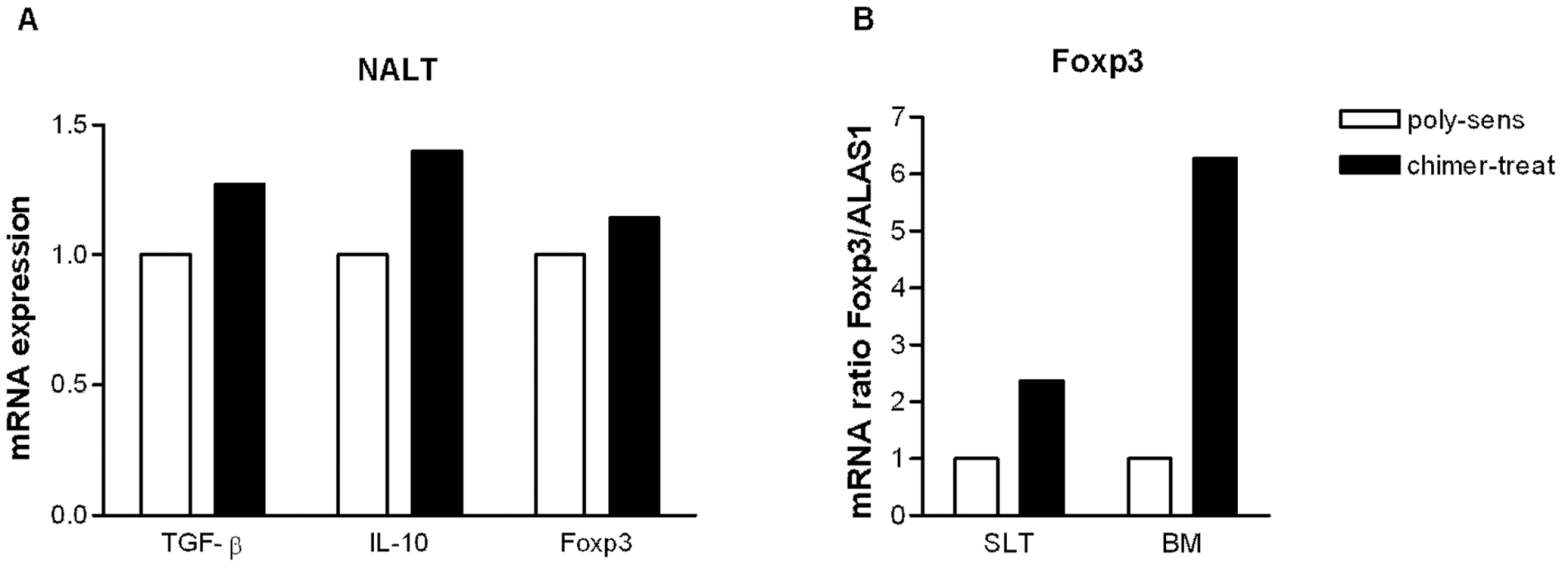
mRNA expression levels of regulatory markers on inductive and local effector sites. (**A**) TGF-β, IL-10 and Foxp3 mRNA expression in NALT, and (**B**) Foxp3 mRNA expression in SLT and BM of chimer-pretreated mice (black bars), shown as relative values in comparison with poly-sensitized controls (white bars). Data are presented as relative ratio of the target genes to the housekeeping gene *Alas1*.

The mRNA expression of TGF-β, IL-10 and Foxp3 in NALT was enhanced in chimer-pretreated mice compared to poly-sensitized controls (Fig. 6A). Increased Foxp3 expression was detected in SLT and BM of chimer-pretreated mice (Fig. 6B).

**Figure 7:**
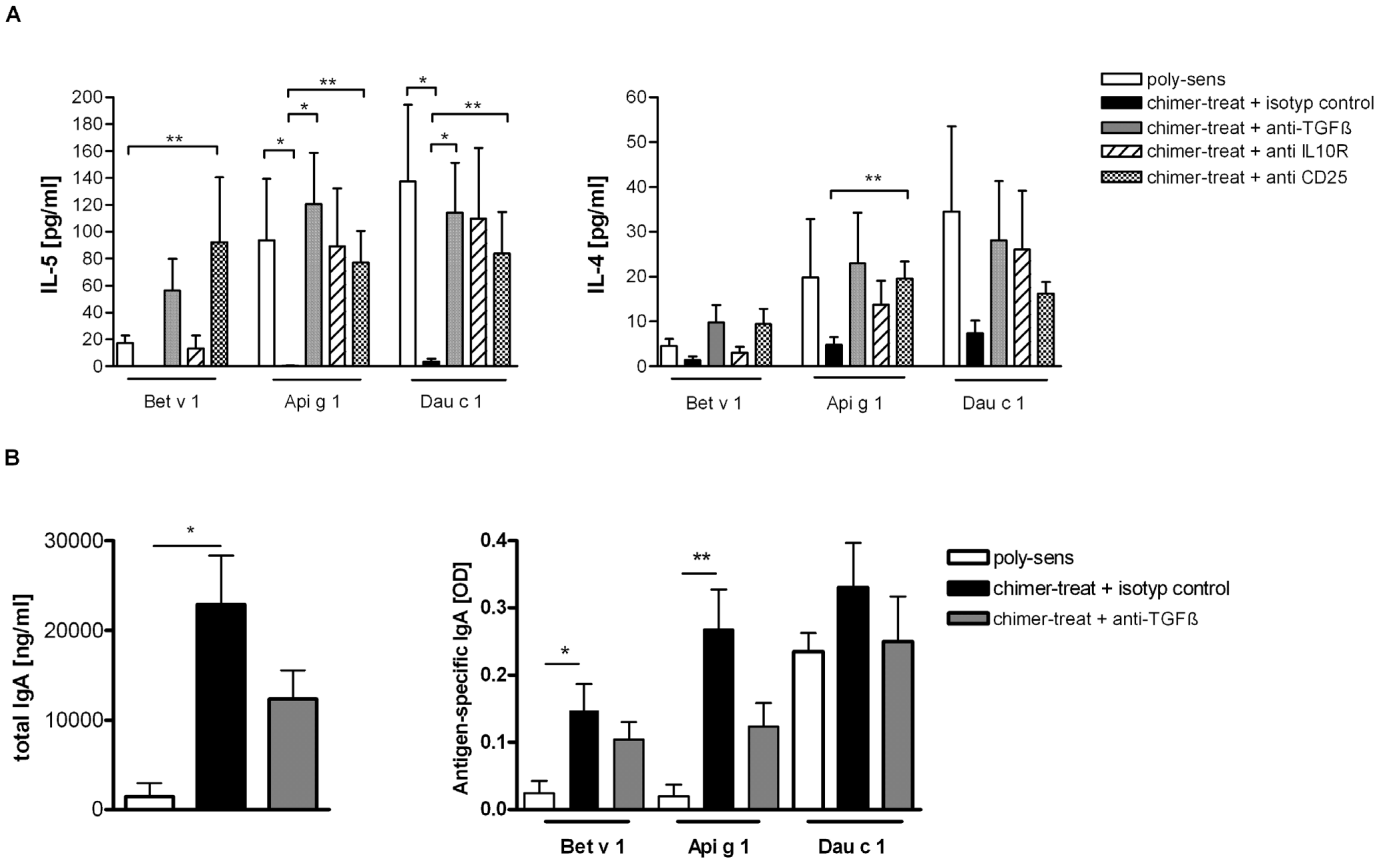
Effects of blocking antibodies. (**A**) Effects of anti-TGF-β, anti-IL-10R and anti-CD25 on levels of IL-5 and IL-4 in supernatants of antigen-stimulated spleen cell cultures. (**B**) Effects of anti-TGF-β on total and antigen-specific IgA production in sera. Chimer-pretreated mice treated with isotype control antibody (black bars) in comparison with chimer-pretreated and anti-TGF-β treated mice (grey bars), anti-IL10R treated mice (striped bars) and anti-CD25 treated mice (dotted bars). Poly-sensitized control group (white bars). *p<0.05, **p<0.01.

Application of anti-TGF-β, anti-IL10R or anti-CD25 blocking antibodies significantly abrogated the suppression of IL-5 and IL-4 production in spleen cell cultures of chimer-treated mice (Fig. 7A). Furthermore anti-TGF-β treatment of chimer-treated mice led to diminished antigen-specific and total IgA levels in sera (Fig. 7B).

**Figure 8:**
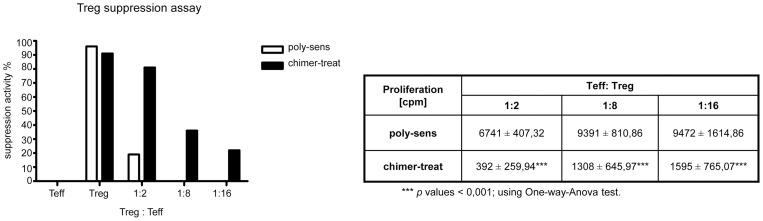
Characterization of Treg cells by Treg suppression assay. Suppressive activity of Treg cells derived from chimer-treated mice (black bars) and poly-sensitized controls (white bars). Purified CD4^+^CD25^−^ T effector cells (Teff) and CD4^+^CD25^+^ T regulatory cells (Treg) were obtained by cell sorting (MACS), then cultured alone or cocultured in three different ratios (Treg:Teff: 1∶2, 1∶8, 1∶16) in combination with irradiated splenocytes and stimulated by anti-CD3 antibody, before pulsing with [^3^H] thymidine. The percent suppression mediated by Treg cells is calculated by the following formula: [(cpm of Teff alone – cpm of Teff treated with Treg)/cpm of Teff cells alone]*100.

Treg cells isolated from chimer-treated mice exhibited stronger suppressive potential as Treg cells from poly-sensitized mice (Fig. 8). The percent suppression mediated by Treg cells isolated from chimer-treated mice was 4.8-fold higher (ratio 1/2) than Tregs derived from poly-sensitized mice.

## Discussion

Clinical data regarding the efficacy of specific immunotherapy (SIT) with BP against pollen-related food allergies provide controversial results: While it has been shown that SIT with BP extract could achieve good effects in mono-sensitized patients [23], SIT in multi-sensitized patients with BPRFA often provided only limited success on the food-related symptoms [6], [7], [24]. Bucher et al. attributed this observation to the reason that BPRFA is not only caused by Bet v 1-cross reactive allergens, but also due to other less well-defined cross-reactive food allergens, which are not included in BP extracts used for SIT [2]. Along these lines, Bohle et al. described the existence of exclusive food-specific T lymphocytes, additionally to BP-reactive T cells in pollen-food multi-sensitized patients and suggested this finding to be one of the reasons why allergic symptoms of pollen-related food allergens are not modulated by BP SIT [1].

Here we describe a murine model for BPRFA which enables us to study the role of both IgE and T cells responses in sensitization to Bet v 1 and Bet v 1-related food allergens as well as the efficacy of prevention of multi-sensitization using a novel allergen chimer covering the major T cell epitopes of Bet v 1, Dau c 1 and Api g 1 (Fig. 1, Fig. 3A).

In order to mimic the clinical situation of BPRFA with OAS, mice were challenged sublingually with pollen and food extracts. Our data show that the sensitization protocol led to systemic Th2-biased immune responses as well as local immune responses to Bet v 1, Api g 1 and Dau c 1. Similarly as seen in humans, we observed that mucosal application of the single allergens, in particular with Bet v 1, did not reduce the allergic responses to the related food allergens indicating that other than Bet v 1 epitopes are necessary for successful prevention of BPRFA (Fig. 2A). Furthermore, a mixture of the allergens also did not suppress the immune responses to all allergens (Fig. 2B). A similar negative interference between several protein allergens was previously described by us, when intranasally applying a mixture of birch and grass pollen allergens aiming to prevent allergic poly-sensitization to these allergens [8]. The failure to prevent allergic poly-sensitization with the allergen mixture pointed out the necessity for creating multi-allergen constructs.

Therefore, we engineered a pollen-food chimer, composed of the Bet v 1 protein as scaffold for linkage of the immunodominant T cell epitopes of Api g 1 and Dau c 1 (Fig. 3A). Of notice, these immunodominant T cell epitopes in mice are located in regions of the dominant T cell sequences of humans with BPRFA [22], [25].

Mucosal application of the chimer led to a marked immunomodulation characterized by a shift towards Th1 immune responses (increase in IgG2a) accompanied by a significant down-regulation of allergen-specific IgE to all three allergens (Fig. 4A/B). In accordance, reduced antigen-specific IL-4 and IL-5 levels versus significantly enhanced antigen-specific IFN-γ levels were observed in restimulated spleens and CLNs after chimer pretreatment (Fig. 5). These findings are in line with our previous studies in poly-sensitized mice using either poly-peptides, hybrid peptides or allergen chimers for prevention of poly-sensitization [8], [10]. Additionally, intranasal pretreatment with the chimer increased IgA antibody levels primarily in sera (Fig. 4C) and less at the mucosal sites (data not shown). This might be explained by a matter of increased systemic circulation due to the highly vascularized tissue of the oral cavity, as it has been suggested in humans [26]. Studies by Pilette et al. showed that successful SIT positively correlates with increased serum IgA antibodies [27]. There is evidence that TGF-β plays a major role in IgA production [28], [29]. Indeed, we have shown that the application of anti-TGF-β antibody to chimer-treated mice reduced IgA levels in sera (Fig. 7B).

Regarding the mechanisms of chimer-induced immunomodulation, we detected an upregulation of TGF-β, IL-10 and Foxp3 mRNA levels in the NALT (Fig. 6A). Additionally, increased Foxp3 mRNA expression occurred in sublingual tissue and the buccal mucosa (Fig. 6B). This is in line with our former data showing that immunomodulation with a grass-birch pollen chimer but also with Bet v 1 alone is mediated by Treg cells [8], [9]. The clinical relevance of this finding is supported by human studies showing an increase of regulatory Foxp3-positive T cells in the nasal mucosa of patients after successful systemic as well as sublingual grass pollen SIT [30], [31]. In the latter study it was suggested that regulatory T cells either migrate from draining lymph nodes to the sublingual tissue during SLIT or may be induced by local dendritic cell-T cell interactions after repeated allergen exposure via the oral mucosa [31]. Furthermore, it was shown that sublingual treatment with allergens led to induction of IL-10 and TGF-β-releasing cells within the oral and nasal mucosa [31].

In order to investigate the immunosuppressive capacity of chimer-induced regulatory T cells in our BPRFA model, neutralizing antibodies against CD25^+^ T cells, TGF-β and IL-10R were injected after mucosal application of the chimer and/or poly-sensitization in mice (Fig. 7A). Indeed, each of these antibodies significantly abrogated the suppressive effect of the chimer pretreatment, indicating that CD25^+^-T cells, TGF-β as well as IL-10 play a potential role in immunomodulation for the prevention of BPRFA. Consistent with this, a study in house dust mite allergic patients showed that application of IL-10 and TGF-β blocking antibodies *in vitro* abrogated the immunosuppressive effects of CD4^+^CD25^+^-T cells induced by SIT [32].

Allergic diseases have been linked with deficiency in function of Tregs [33]. Indeed we have shown that chimer-induced Tregs have higher suppression potential in comparison to Tregs derived from poly-sensitized mice (Fig. 8).

Taken together, we constructed an allergen-chimer for prevention of multiple sensitizations to pollen and pollen-related food allergens. We demonstrated that mucosally applied chimers covering important pollen and food-related epitopes can be used to down-regulate/prevent systemic and local allergic immune responses to all allergens, most likely by a combined induction of regulatory pathways and Th1-biased immune responses. Such mucosal allergen constructs might therefore provide promising new tools for mucosal intervention against different levels of multi-sensitization, including the birch pollen-related food allergy.

## References

[1] Bohle B (2007). The impact of pollen-related food allergens on pollen allergy.. Allergy.

[2] Bucher X, Pichler WJ, Dahinden CA, Helbling A (2004). Effect of tree pollen specific, subcutaneous immunotherapy on the oral allergy syndrome to apple and hazelnut.. Allergy.

[3] Hoffmann-Sommergruber K (2000). Plant allergens and pathogenesis-related proteins. What do they have in common?. Int Arch Allergy Immunol.

[4] Steinman H (2009). Oral allergy syndrome - whats new?. Current Allergy & Clinical Immunology 22.

[5] Geroldinger-Simic M, Zelniker T, Aberer W, Ebner C, Egger C (2011). Birch pollen-related food allergy: clinical aspects and the role of allergen-specific IgE and IgG4 antibodies.. J Allergy Clin Immunol 127: 616–622 e611.

[6] Hansen KS, Khinchi MS, Skov PS, Bindslev-Jensen C, Poulsen LK (2004). Food allergy to apple and specific immunotherapy with birch pollen.. Mol Nutr Food Res.

[7] Mauro M, Russello M, Incorvaia C, Gazzola G, Frati F (2011). Birch-apple syndrome treated with birch pollen immunotherapy.. Int Arch Allergy Immunol.

[8] Wild C, Wallner M, Hufnagl K, Fuchs H, Hoffmann-Sommergruber K (2007). A recombinant allergen chimer as novel mucosal vaccine candidate for prevention of multi-sensitivities.. Allergy.

[9] Winkler B, Hufnagl K, Spittler A, Ploder M, Kallay E (2006). The role of Foxp3+ T cells in long-term efficacy of prophylactic and therapeutic mucosal tolerance induction in mice.. Allergy.

[10] Hufnagl K, Winkler B, Focke M, Valenta R, Scheiner O (2005). Intranasal tolerance induction with polypeptides derived from 3 noncross-reactive major aeroallergens prevents allergic polysensitization in mice.. J Allergy Clin Immunol.

[11] Bublin M, Radauer C, Wilson IB, Kraft D, Scheiner O (2003). Cross-reactive N-glycans of Api g 5, a high molecular weight glycoprotein allergen from celery, are required for immunoglobulin E binding and activation of effector cells from allergic patients.. Faseb J.

[12] Wiedermann U, Jahn-Schmid B, Bohle B, Repa A, Renz H (1999). Suppression of antigen-specific T- and B-cell responses by intranasal or oral administration of recombinant bet v 1, the major birch pollen allergen, in a murine model of type I allergy.. J Allergy Clin Immunol.

[13] Hufnagl K, Wagner B, Winkler B, Baier K, Hochreiter R (2003). Induction of mucosal tolerance with recombinant Hev b 1 and recombinant Hev b 3 for prevention of latex allergy in BALB/c mice.. Clin Exp Immunol.

[14] Leech MD, Benson RA, De Vries A, Fitch PM, Howie SE (2007). Resolution of Der p1-induced allergic airway inflammation is dependent on CD4+CD25+Foxp3+ regulatory cells.. J Immunol.

[15] Wilson MS, Taylor MD, Balic A, Finney CA, Lamb JR (2005). Suppression of allergic airway inflammation by helminth-induced regulatory T cells.. J Exp Med.

[16] Taher YA, van Esch BC, Hofman GA, Henricks PA, van Oosterhout AJ (2008). 1alpha,25-dihydroxyvitamin D3 potentiates the beneficial effects of allergen immunotherapy in a mouse model of allergic asthma: role for IL-10 and TGF-beta.. J Immunol.

[17] Wiedermann U, Herz U, Baier K, Vrtala S, Neuhaus-Steinmetz U (2001). Intranasal treatment with a recombinant hypoallergenic derivative of the major birch pollen allergen Bet v 1 prevents allergic sensitization and airway inflammation in mice.. Int Arch Allergy Immunol.

[18] Asanuma H, Thompson AH, Iwasaki T, Sato Y, Inaba Y (1997). Isolation and characterization of mouse nasal-associated lymphoid tissue.. J Immunol Methods.

[19] Cuburu N, Kweon MN, Song JH, Hervouet C, Luci C (2007). Sublingual immunization induces broad-based systemic and mucosal immune responses in mice.. Vaccine.

[20] Winkler B, Baier K, Wagner S, Repa A, Eichler HG (2002). Mucosal tolerance as therapy of type I allergy: intranasal application of recombinant Bet v 1, the major birch pollen allergen, leads to the suppression of allergic immune responses and airway inflammation in sensitized mice.. Clin Exp Allergy.

[21] Hufnagl K, Focke M, Gruber F, Hufnagl P, Loupal G (2008). Airway inflammation induced after allergic poly-sensitization can be prevented by mucosal but not by systemic administration of poly-peptides.. Clin Exp Allergy.

[22] Jahn-Schmid B, Radakovics A, Luttkopf D, Scheurer S, Vieths S (2005). Bet v 1142–156 is the dominant T-cell epitope of the major birch pollen allergen and important for cross-reactivity with Bet v 1-related food allergens.. J Allergy Clin Immunol.

[23] Asero R (1998). Effects of birch pollen-specific immunotherapy on apple allergy in birch pollen-hypersensitive patients.. Clin Exp Allergy.

[24] Walker SM, Durham SR, Till SJ, Roberts G, Corrigan CJ (2011). Immunotherapy for allergic rhinitis.. Clin Exp Allergy.

[25] Bohle B, Radakovics A, Jahn-Schmid B, Hoffmann-Sommergruber K, Fischer GF (2003). Bet v 1, the major birch pollen allergen, initiates sensitization to Api g 1, the major allergen in celery: evidence at the T cell level.. Eur J Immunol.

[26] Moingeon P, Batard T, Fadel R, Frati F, Sieber J (2006). Immune mechanisms of allergen-specific sublingual immunotherapy.. Allergy.

[27] Pilette C, Nouri-Aria KT, Jacobson MR, Wilcock LK, Detry B (2007). Grass pollen immunotherapy induces an allergen-specific IgA2 antibody response associated with mucosal TGF-beta expression.. J Immunol.

[28] Sonoda E, Matsumoto R, Hitoshi Y, Ishii T, Sugimoto M (1989). Transforming growth factor beta induces IgA production and acts additively with interleukin 5 for IgA production.. J Exp Med.

[29] Coffman RL, Lebman DA, Shrader B (1989). Transforming growth factor beta specifically enhances IgA production by lipopolysaccharide-stimulated murine B lymphocytes.. J Exp Med.

[30] Radulovic S, Jacobson MR, Durham SR, Nouri-Aria KT (2008). Grass pollen immunotherapy induces Foxp3-expressing CD4+ CD25+ cells in the nasal mucosa.. J Allergy Clin Immunol 121: 1467–1472, 1472 e1461.

[31] Scadding GW, Shamji MH, Jacobson MR, Lee DI, Wilson D (2010). Sublingual grass pollen immunotherapy is associated with increases in sublingual Foxp3-expressing cells and elevated allergen-specific immunoglobulin G4, immunoglobulin A and serum inhibitory activity for immunoglobulin E-facilitated allergen binding to B cells.. Clin Exp Allergy.

[32] Jutel M, Akdis M, Budak F, Aebischer-Casaulta C, Wrzyszcz M (2003). IL-10 and TGF-beta cooperate in the regulatory T cell response to mucosal allergens in normal immunity and specific immunotherapy.. Eur J Immunol.

[33] Verhagen J, Akdis M, Traidl-Hoffmann C, Schmid-Grendelmeier P, Hijnen D (2006). Absence of T-regulatory cell expression and function in atopic dermatitis skin.. J Allergy Clin Immunol.

